# The Effect of Tau and Taxol on Polymerization of MCF7 Microtubules In Vitro

**DOI:** 10.3390/ijms23020677

**Published:** 2022-01-08

**Authors:** Mitra Shojania Feizabadi, Venise Jan Castillon

**Affiliations:** Department of Physics, Seton Hall University, South Orange, NJ 07079, USA; venisejan.castillon@student.shu.edu

**Keywords:** microtubule, MCF7 cell, Tau protein, Taxol, tubulin isotypes, drug resistance

## Abstract

Overexpression of Tau protein in breast cancer cells is identified as an indicator for potential resistance to taxane-based therapy. As reported findings have been obtained mostly from clinical studies, the undetermined underlying mechanism of such drug resistance needs to be thoroughly explored through comprehensive in vitro evaluations. Tau and Taxol bind to the beta tubulin site in microtubules’ structure. This is of particular interest in breast cancer, as microtubules of these cancer cells are structurally distinct from some other microtubules, such as neuronal microtubules, due to their unique beta tubulin isotype distribution. The observed changes in the in vitro polymerization of breast cancer microtubules, and the different function of some molecular motors along them, leave open the possibility that the drug resistance mechanism can potentially be associated with different responses of these microtubules to Tau and Taxol. We carried out a series of parallel experiments to allow comparison of the in vitro dual effect of Tau and Taxol on the polymerization of MCF7 microtubules. We observed a concentration-dependent demotion-like alteration in the self-polymerization kinetics of Tau-induced MCF7 microtubules. In contrast, microtubules polymerized under the simultaneous effects of Tau and Taxol showed promoted assembly as compared with those observed in Tau-induced microtubules. The analysis of our data obtained from the length of MCF7 microtubules polymerized under the interaction with Tau and Taxol in vitro suggests that the phenomenon known as drug resistance in microtubule-targeted drugs such as Taxol may not be directly linked to the different responses of microtubules to the drug. The effect of the drug may be mitigated due to the simultaneous interactions with other microtubule-associated proteins such as Tau protein. The observed regulatory effect of Tau and Taxol on the polymerization of breast cancer microtubules in vitro points to additional evidence for the possible role of tubulin isotypes in microtubules’ functions.

## 1. Introduction

Microtubules (MTs), one of the intracellular filaments, contribute to diverse cellular functions including cell division, cell shape, and intracellular transportation. These dynamic bio-filaments are structured from α- and β-tubulin heterodimers. They interact with microtubule-associated proteins (MAPs) as well as microtubule-targeting agents such as Taxol which binds to a site on the β-tubulin of a microtubule [1,2,3]. Other studies showed that Tau protein, one of the microtubule-associated proteins, also binds to the microtubule through the same β-tubulin binding site as Taxol [4,5,6]. In vitro studies on neuronal microtubules confirmed that Taxol enhances microtubule assembly [7,8]. Similarly, in vitro studies on the interaction of Tau protein with neuronal microtubules showed that Tau protein also promotes the polymerization and stabilization of neuronal microtubules [9,10,11]. Among these extensive studies, a study by Panda et al. reported the regulatory effect of Tau protein on the dynamics of neuronal microtubules in vitro by thoroughly evaluating the role which different isoforms of Tau protein can play in altering microtubules’ dynamics. In this study, knowing that human brain Tau protein consists of six different isoforms, they showed that Tau protein with some specific isoforms binds more strongly to neuronal microtubules and causes greater stability as compared with different isoforms—confirmation of a correlation between the underlying functional mechanism of Tau protein and its compositional structure [12]. Furthermore, the presence of different isoforms of Tau protein in non-neuronal cells has been observed, which, together with the study by Panda et al., supports a relationship between the source of Tau protein and its regulatory role in microtubules [13].

Intriguingly, diverse tubulin isotypes that exist in different cells lead to the formation of structurally distinct microtubules in cells of different tissues [14]. Extended reported studies revealed that observed changes in the dynamics of some microtubules and translocation characteristics of some molecular motors along microtubules can be potentially isotype specific [15,16,17,18,19].

The correlation between the critical role of tubulin isotypes and the distinct intracellular functions of some cells strengthens the possibility that some of the open questions regarding cancer and the associated treatments may also be linked to the unique response to tubulin isotypes while interacting with anti-cancer agents and intracellular proteins. This can be especially important in studying the interaction of Taxol and Tau protein in breast cancer microtubules to identify the underlying mechanism that leads to the taxane resistance observed in breast cancer cells with overexpression of Tau protein [20,21].

Breast cancer (MCF7) microtubules are composed of 0% β II, 39.1% β I, 2.5% β III, and 58.4% β IV. This distribution of beta tubulin isotypes distinguishes them from neuronal microtubules with 3% βI, 58% β II, 25% β III, and 13% β VI in their structure [14]. Previous studies have confirmed that both Tau protein, which is widely expressed in neuronal cells, and Taxol bind to beta tubulin sites, stabilize, and promote the polymerization of neuronal microtubules [5]. In breast cancer cells, a similar response in microtubules’ polymerization is observed when they interact with Taxol. However, the overexpression of Tau protein in some breast cancer cells is known as an indicator to predict the potential resistance to taxane treatment [22,23]. Nevertheless, the associated underlying mechanism is still poorly known.

In our prior work, we reported a regulatory effect of Tau protein on the polymerization of MCF7 microtubules in vitro, where no evidence of a polymerization-promoting effect was observed [24]. As the study of Bhandare et al. confirmed the different binding affinity of Tau protein to different beta tubulin isotypes, the change in kinetic specifications, including the stability and growth of MCF7 microtubules in the presence of Tau protein, may be associated with a different interaction mechanism of this protein with MCF7 microtubules [25].

Motivated by the need to more clearly understand the complexity of drug resistance in breast cancer cells and possible underlying mechanisms, we studied the effect of the dual interaction of Tau and Taxol on the assembly and polymerization of MCF7 microtubules in vitro through comparing the length of MCF7 microtubules in parallel experiments. The uniqueness of this study is in the direct assessment of the possible changes that can be induced in instantaneous polymerization specifications of human breast cancer tubulin in the absence of any other cellular elements, which can possibly contribute to understanding the drug resistance mechanism in breast cancer cells with a high level of Tau proteins.

## 2. Results

**Figure 1:**
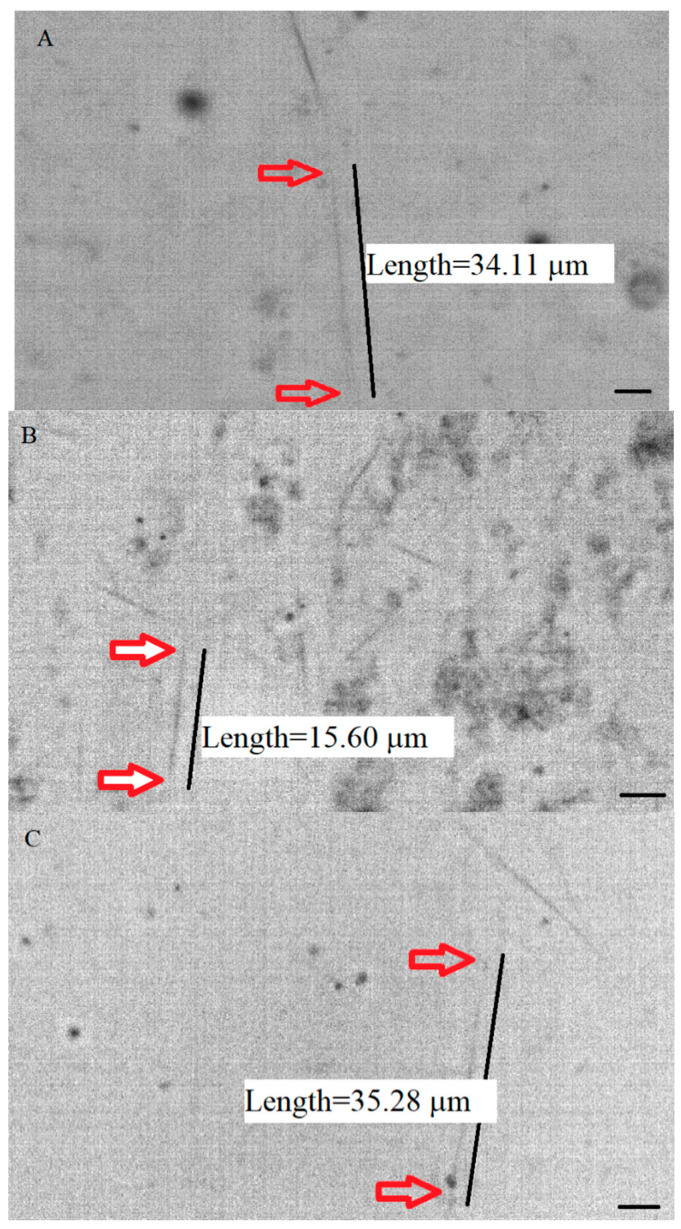
Samples of pictures taken with a dark-field microscope. For better visibility, the contrast of the images is inverted in the above pictures. In the pictures, the individual microtubule polymerized from (**A**) pure MCF7 tubulin, (**B**) MCF7 tubulin and Tau protein with Tau/tubulin ratio equal to 0.1, and (**C**) MCF7 tubulin and Tau protein with Tau/tubulin ratio equal to 0.1, and Taxol with the concentration of 0.2 µM, is identified. The concentration of MCF7 tubulin was 2 mg/mL. The scale bar is 5 µm.

We developed a dark-field video microscopy method to visualize and analyze the assembly of individual breast cancer microtubules under the dual effect of Tau protein and Taxol. The data were obtained from microtubules polymerized from three groups: (A) pure MCF7 tubulin with the total concentrations of 1.25, 2, and 2.5 mg/mL, (B) a mixture of MCF7 tubulin with the final concentrations listed in group A above and Tau protein with a final concentration equal to 10% of the concentration of tubulin in the mixture, and (C) a mixture similar to that categorized in group B with 0.2 µM of Taxol. Figure 1 shows samples of images obtained from the dark-field microscopy of polymerized microtubules.

**Figure 2:**
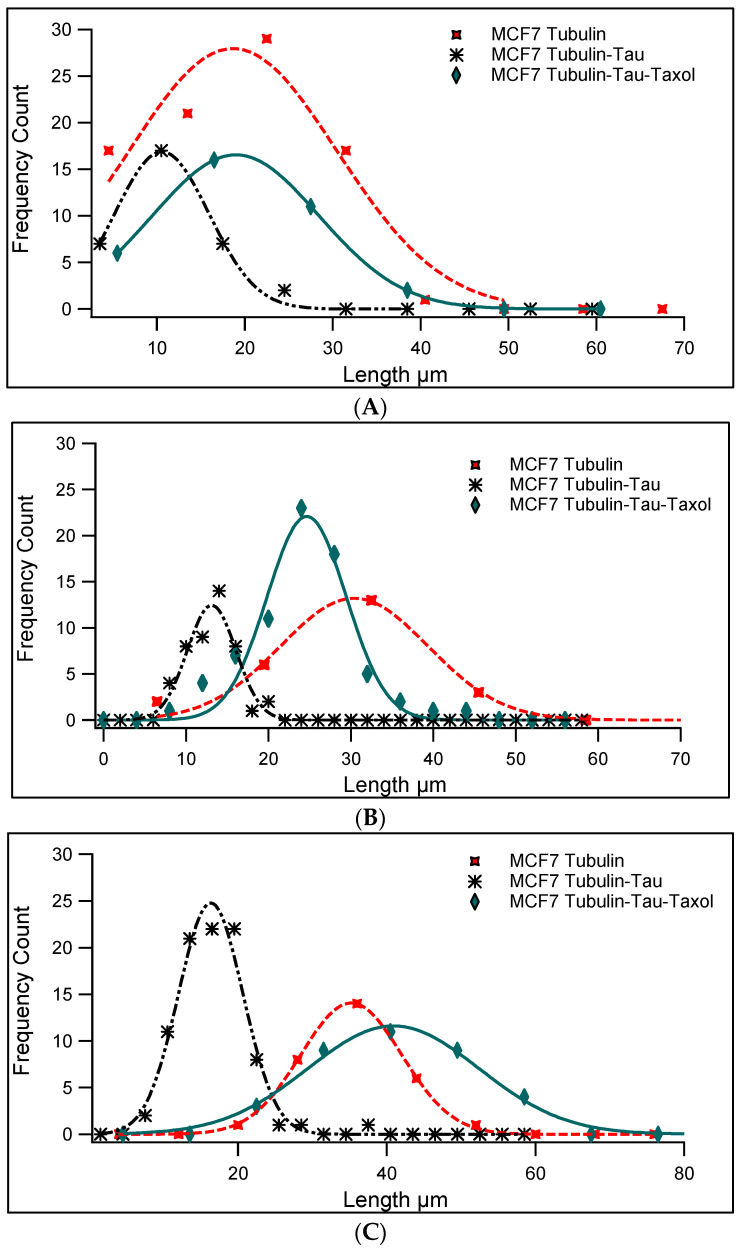
Histograms of microtubules’ lengths. The average lengths were calculated via Gaussian curve fitting. (**A**) represents marker histograms obtained from a concentration of 1.25 mg/mL of tubulin, 0.1 mg/mL of Tau, and 0.2 µM of Taxol. The average length of microtubules from MCF7 microtubules was 18.7 ± 8.4 µm (*n* = 85, red, width = 16.8 µm); Tau-induced MTs’ average length at this concentration was 10.6 ± 3.8 µm (*n* = 33, black, width = 7.6 µm); and Tau and Taxol-induced MTs’ average length was 19.0 ± 6.7 µm (*n* = 35, green, width = 13.4 µm). A t-test was used to compare the average lengths between two groups of polymerized microtubules (MCF7 and MCF7-Tau, and MCF7-Tau and MCF7-Tau-Taxol). The average lengths were significantly different (*p* = 0.0001, *p* = 0.0001). (**B**) In the samples obtained from 2 mg/mL of tubulin, 0.2 mg/mL of Tau, and 0.2 µM of Taxol, the average length of MCF7 microtubules was 30.4 ± 6.3 µm (*n* = 24, red, width = 12.6 µm); Tau-induced MTs’ average length at this concentration was 13.1 ± 2.1 µm (*n* = 46, black, width = 4.2 µm); and Tau and Taxol-induced MTs’ average length was 24.6 ± 3.4 µm (*n* = 73, green, width = 6.8 µm). The average lengths between the two groups mentioned above were significantly different (*p* = 0.0001, *p* = 0.0001). (**C**) In samples consisting of 2.5 mg/mL of tubulin, 0.25 mg/mL of Tau, and 0.2 µM of Taxol, the average length of microtubules from MCF7 microtubules was 35.2 ± 4.8 µm (*n* = 30, red, width = 9.6 µm); Tau-induced MTs’ average length at this concentration was 16.3 ± 3.1 µm (*n* = 89, black, width = 6.2 µm); and Tau and Taxol-induced MTs’ average length was 40.9 ± 8.1 µm (*n* = 36, green, width = 16.2). Similar to the two other cases, the average lengths were significantly different (*p* = 0.0001, *p* = 0.0001).

**Figure 3:**
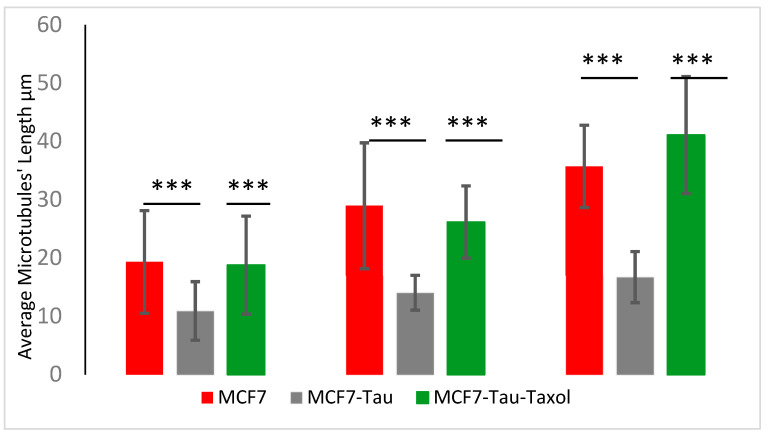
The differences between the average lengths obtained from the raw data of microtubules polymerized from pure tubulin (red), a mixture of tubulin and Tau (gray), and a mixture of tubulin, Tau, and Taxol (green) were statistically compared through a *t*-test. The differences were all statistically significant at different concentrations, with a *p* value less than 0.0001. The (***) represents the significant difference between two mean values.

When several lengths of individual microtubules in different samples were analyzed and measured, we were able to create a histogram of the microtubules’ lengths. From these histograms, via Gaussian fitting, we obtained the average length of the microtubules (Figure 2). In addition, the obtained average lengths from the raw data are presented in Table 1 and statistically analyzed in Figure 3.

First, the sole effect of Taxol on MCF7 polymerization was tested in samples with the minimum tubulin concentration of 1.25 mg/mL. The average length of microtubules polymerized in the presence of 0.2 µM of Taxol from the raw data was found to be 27.6 ± 4.0 µm (*n* = 13), with no significant change in the population of MTs in the field of view. Compared with the average length of MTs polymerized from pure tubulin at the same concentration (19.3 ± 8.8 µm, *n* = 85), the significant difference in the average length is aligned with the reported overall stabilizing effect of Taxol in the polymerization of microtubules [26].

Second, the observed increase in the microtubules’ length indicates that the polymerization of microtubules from pure MCF7 tubulin is sensitive to the concentration of free tubulin (Table 1). The differences between the average lengths obtained at the three different concentrations were significantly different from one another (*p* = 0.0001). We and others previously reported that some cancer microtubules, including MCF7, show slow and stable dynamics where they seldom switch to the depolymerization phase [27,28]. Therefore, the observed increase in the length can be mainly associated with the growth rate of MCF7 microtubules and consequently be directly proportional to the concentration of free tubulin subunits, an observation consistent with a previously reported study on neuronal microtubules in vitro [29].

Third, the sole effect of Tau protein in the polymerization of MCF7 MTs was examined. In the samples of Tau-induced polymerized MCF7 microtubules, a 50% reduction in the average microtubule lengths polymerized at different tubulin concentrations was observed. The difference in the length obtained from microtubules polymerized in the presence and absence of Tau protein was statistically significant (*p* = 0.0001). This suggests that the presence of Tau protein with a concentration equal to 10% of the tubulin concentration changes the kinetics of self-assembly of MCF7 microtubules. To further analyze this observed change, the number of MTs in the field of view was estimated in several samples with two tubulin concentrations of 1.25 mg/mL and 2 mg/mL. The average estimated number of assembled microtubules from 1.25 mg/mL of pure MCF7 tubulin was 3.4 (the number of samples analyzed, *n* = 20), and in the presence of Tau protein, this number was 7.3 (*n* = 16). In samples of microtubules polymerized from 2 mg/mL of MCF7 tubulin, these numbers were consequently equal to 5.2 (*n* = 21) and 9.3 (*n* = 16).

As observed, the increase in the number of MTs in the presence of Tau protein, along with the formation of shorter MCF7 microtubules, suggests that the alteration in the self-polymerization mechanism can partially be associated with the enhancer effect of Tau protein in the spontaneous nucleation of MCF7 microtubules. However, the observed length differences can be caused by the change in the dynamic parameters of microtubules. In our previous study, a change in Tau-induced microtubules at a certain concentration was observed when a “seed method” was utilized. In that method, microtubules were directly polymerized from the mixture of Tau and Taxol in samples prepared on microscope slides. This is also an indication that it is less probable that the shorter observed length is linked to microtubule breakage during sample preparation [24].

As MCF7 microtubules show slow and stable dynamics, the change may be due to the change in the rate of growth of MTs in the presence of Tau protein; this is an open question which needs to be quantified in future studies. This can be accomplished by immobilizing one end of a single microtubule through polymerization induced by seeds in the presence of a low concentration of tubulin.

Fourth, the observation procedure was conducted in the group of samples prepared from the mixtures of Tau and Taxol with tubulin in different concentrations. We observed a significant increase in the length of microtubules as compared with Tau-induced microtubules. This difference in length was statistically significant (*p* = 0.0001). In samples of MTs with both Tau protein and Taxol, polymerized from MCF7 tubulin with concentrations of 1.25 mg/mL and 2 mg/mL, the average estimated number of MTs was 5.5 (*n* = 17) and 8.3 (*n* = 26), respectively. The addition of Taxol did not significantly change the estimated number of MTs but caused a significant increase in MTs’ lengths, which is aligned with the stabilizing effect of Taxol in the polymerization of microtubules.

Finally, we repeated parallel experiments to assess the effect of different concentrations of Tau protein on MCF7 microtubules’ assembly. We examined the microtubules polymerized from the mixture of MCF7 tubulin with concentrations of 2.5 mg/mL and 0.1 mg/mL of Tau protein (almost 5% of the concentration of tubulin). The average length was found to be 29.3 ± 4.7 µm (*n* = 52). While a slight reduction was observed as compared with MTs polymerized from pure MCF7 tubulin at the same concentration with an average length of 35.2 ± 0.1 µm, the difference in length was statistically insignificant (*p* = 0.35). Collective analysis of the average length of MTs interacting with Tau protein suggested that the interacting response of MCF7 tubulin and Tau protein depends on the Tau/tubulin ratio. While the change in length of the polymerized MTs was not significant in the presence of a low concentration of Tau protein, it became noticeable as the concentration of this protein increased. These results were obtained under our choice of concentrations for Tau, Taxol, and tubulin; however, the dependency of the results on the concentration variations of Tau and Taxol should be the subject of more extensive future studies.

## 3. Discussion

The efficacy of taxane-containing therapy in patients with breast cancer is multi-factorial. In several clinical studies, the level of Tau in patients is identified as a biomarker for predicting the outcome of treatment with taxane [22]. As observed in our previous study and reported by others as well, in the polymerization of neuronal microtubules in vitro, Tau protein catalyzes the self-assembly of microtubules and decreases the rate of depolymerization [9,10,24]. Additionally, Taxol is known as a polymerization promoter because of its stabilizing effect on microtubules’ growth.

While both Tau and Taxol showed enhancing effects on the polymerization of neuronal microtubules in vitro, they showed different effects on the polymerization of MCF7 microtubules in vitro in our current study. Tau protein consists of three domains; the C-terminal domain, which is positively charged, binds to the beta tubulin of microtubules and promotes the assembly of neuronal MTs [30,31]. As the C-terminal tail of beta tubulin isotypes has been reported to carry different values of negative charge, the Tau–tubulin conformation may be affected due to different electrostatic interactions [24,32]. In addition, binding of Tau to tubulin is regulated by post-translational modifications, which may even neutralize the positive charge of Tau protein and consequently detach Tau from microtubules [30,33,34,35]. Some studies have shown a possibility that neuronal cells choose how Tau is post-translationally modified [30,36]. It is reported that the post-translational modification of Tau protein as well as the binding of Tau protein to microtubules also depends on Tau isoforms. Tau protein has six different isoforms in the human brain with distinct numbers of post-translational modification sites [12,36]. Therefore, the levels of isoforms in different sources of Tau protein may contribute in different ways, in which they undergo distinct post-translational modifications and perform their overall regulatory roles.

Referring to such ongoing research, there is also a possibility that different post-translational modifications of Tau in different cells cause different Tau–tubulin conformations and consequently alter their regulatory effect on the assembly and dynamics of microtubules; this is a subject that needs further decoding and investigation.

In addition, beta tubulin is the Taxol binding site as well [37,38]. While the M-loop is reportedly involved in drug binding, differences in some of beta tubulin’s residues have been suggested as another factor that affects drug binding [39]. Although further investigation is needed to ascertain the fine lines that shed light on the molecular interaction differences between Tau and Taxol with microtubules of different cells, the current growing evidence demonstrates that the beta tubulin specifications may play a key role in such interactions.

Our results suggest a tug-of-war interpretation of the dual effect of Tau and Taxol on MCF7 microtubules’ polymerization. As the uniqueness of the tubulin isotype distribution in MCF7 microtubules makes our in vitro study distinct from prior studies on neuronal microtubules, establishing a connection between the observed polymerization behavior and the role of tubulin isotypes is appealing and is the subject of many studies. Based on previously reported results, which have been mainly reflected in the review of Huzil et al., microtubules with different tubulin isotype distributions may respond differently to Taxol. This has been viewed as one mechanism of resistance to Taxol [40]. However, the data obtained in our study are one example which confirms that the drug resistance mechanism may not be exclusively linked to the different responses to the drug. The less successful treatment outcome with Taxol, which is a microtubule-targeted drug with the effect of enhancing the polymerization of microtubules, may be caused by other microtubule-associated proteins such as Tau protein that show distinct regulatory effects while interacting with microtubules with different compositional structures.

Collectively, extending in vitro studies leads to better understanding the fine details, which may be a step toward more comprehensive studies and achieving more effective treatment strategies.

## 4. Materials and Methods

### 4.1. Protein Preparation

We used MCF7 tubulin (Cytoskeleton, Denver, CO, USA, Cat. H005). For Tau protein, we used protein obtained from a bovine brain source (Cytoskeleton, Denver, CO, USA, Cat. TA01).

The major chemical compounds used were General Tubulin Buffer containing BRB80: 80 mM PIPES, pH 7.0, 0.5 mMEGTA, and 1 mM MgCl_2_ (Cytoskeleton, Denver, CO, USA, Cat. BST01), Guanosine triphosphate (GTP) (Cytoskeleton, Denver, CO, USA, Cat. BST06), Taxol (Cytoskeleton, Denver, CO, USA, Cat. TA01), and dimethyl sulfoxide (DMSO). The lyophilized MCF7 tubulin was resuspended in polymerization buffer (containing General Tubulin Buffer and 1.0 mM GTP). In addition, the lyophilized Taxol was resuspended in DMSO to reach a 2 mM concentration. It was then diluted with the polymerization buffer. The final concentration of Taxol in all samples was 0.2 µM.

The prepared protein can be categorized into three groups: (a) MCF7 tubulin, (b) mixture of tubulin and Tau protein, (c) mixture of tubulin, Taxol, and Tau protein.

We conducted experiments with different concentrations of MCF7 tubulin in the samples. The final concentrations of MCF7 tubulin were 1.25 mg/mL, 2 mg/mL, and 2.5 mg/mL. In each group, the final concentration of Tau protein was 10% of the final concentration of tubulin: 0.1 mg/mL, 0.2 mg/mL, and 0.25 mg/mL. The total concentration of Taxol was 0.2 µM in all samples. However, in samples with 2.5 mg/mL of tubulin, we also checked the effect of the lower concentration of Tau (0.1 mg/mL).

In our experiment, to form microtubules, proteins prepared in the three groups explained above were mixed in an Eppendorf tube and were incubated for 5 h at 37 °C. In each experiment, after incubation, multiple “samples” were constructed, as explained below, on microscope slides. Under the same experimental conditions, a couple of experiments were conducted for each of the understudy groups mentioned above. In each experiment, multiple samples of polymerized microtubes were constructed by adding 1–1.2 µL of microtubules on a microscope slide. Through this process, we had access to, and the ability to evaluate, independent samples. To transfer polymerized microtubules, we consistently used medium-sized pipette tips to minimize the possibility of microtubule breakage. Microscope slides were then covered by a clean coverslip (Tedpella, thickness No.0, 12 mm × 12 mm) and sealed. The pressure produced by the surface of the microscope slides prevented the flow of microtubules in the samples. Each sample was then observed under a microscope. To assure the randomness of the measurement and sampling, a maximum of two fields were selected in each prepared sample. The selected fields were significantly separated from one another to eliminate any possible overlapping. Additionally, the selected microtubules had no overlap with each other and were fully placed in the field of view. The number of microtubules that were in a field of view was different, but at minimum, we were able to identify 2–3 microtubules in each selected field of view.

### 4.2. Visualization, Measurements, and Data Analysis

Samples were visualized by a Nikon upright microscope (ECLIPSE-Ci-S/Ci-L) equipped with a 100X/1.25 NA oil immersion objective lens, and a 1.43–1.20 oil dark-field condenser. Samples were visualized by a Lumenera camera (Infinity HD), and the pictures and videos were captured from the field of view (115 µm × 85 µm). The recorded videos were analyzed by ImageJ (Rasband, W. S, ImageJ, National Institutes of Health, Bethesda, MD, USA). By analyzing individual microtubules, data related to the positions of microtubules’ ends were obtained from different samples, and the length of each microtubule was calculated.

To calculate the average length in each group of microtubules, histograms of the lengths were constructed from the lengths calculated from non-normalized datasets using Igor Pro 8.04. The mean and SD of each dataset were calculated after utilizing the curve fitting feature of the program. The statistics feature of the program was employed to apply the t-test. In addition, two datasets were statistically assessed by the Mann–Whitney test or a two-tailed Student t-test (GraphPad) to specifically obtain the *p*-value (Igor Pro does not have the feature to calculate the *p*-value in each dataset). In the comparison of each pair of variables, the threshold for the significance level was 0.05, and a 95% confidence interval was utilized. For the pool of non-normalized raw data, the mean +/− SD was calculated by Microsoft Excel (Excel 365). Knowing the size of the samples (*n* = number of microtubules analyzed) and the mean (+/−SD), Student’s *t*-test (GraphPad) was employed for the statistical analysis.

## Figures and Tables

**Table 1:** The average lengths ± SD of polymerized microtubules obtained from the direct calculations (raw data) of lengths are presented. In Figure 3, these data are statistically analyzed. The average lengths obtained from the curve fitting and raw data are very much compatible and are not statistically significantly different.

Samples (Horizontal) Concentrations (Vertical)	Pure Tubulin MTs’ Average Length	Tau-Induced MTs’ Average Length	Tau and Taxol-Induced MTs’ Average Length
Tubulin and Tau (1.25, 0.1) mg/mL	19.3 ± 8.8 µm,	10.9 ± 5.0 µm,	18.8 ± 8.4 µm,
	*n* = 85	*n* = 33	*n* = 35
Tubulin and Tau (2, 0.2) mg/mL	29.0 ± 10.8 µm,	14.0 ± 3.0 µm,	26.2 ± 6.3 µm,
	*n* = 24	*n* = 46	*n* = 73
Tubulin and Tau (2.5, 0.25) mg/mL	35.7 ± 7.1 µm,	16.7 ± 4.4 µm,	41.1 ± 10.0 µm,
	*n* = 30	*n* = 89	*n* = 36
